# Effect of Herbicide-Resistant Oil-Degrading Bacteria on Plants in Soil Contaminated with Oil and Herbicides

**DOI:** 10.3390/plants13243560

**Published:** 2024-12-20

**Authors:** Tatyana Korshunova, Elena Kuzina, Svetlana Mukhamatdyarova, Milyausha Iskuzhina, Liliya Kulbaeva, Svetlana Petrova

**Affiliations:** 1 Ufa Institute of Biology, Ufa Federal Research Centre, Russian Academy of Sciences, Ufa 450054, Russia; misshalen@mail.ru (E.K.); svetrm@gmail.com (S.M.); ishmurzina82@mail.ru (M.I.); l.kulbaeva78@mail.ru (L.K.); 2 Ufa Institute of Chemistry, Ufa Federal Research Centre, Russian Academy of Sciences, Ufa 450054, Russia; petrova_sf89@anrb.ru

**Keywords:** oil, herbicides, bioremediation, growth-stimulating properties, lupine, oats

## Abstract

Biological remediation of agricultural soils contaminated with oil is complicated by the presence of residual amounts of chemical plant protection products, in particular, herbicides, which, like oil, negatively affect the soil microbiome and plants. In this work, we studied five strains of bacteria of the genera *Pseudomonas* and *Acinetobacter*, which exhibited a high degree of oil biodegradation (72–96%). All strains showed resistance to herbicides based on 2,4-D, imazethapyr and tribenuron-methyl, the ability to fix nitrogen, phosphate mobilization, and production of indole-3-acetic acid. The presence of pollutants affected the growth-stimulating properties of bacteria in different ways. The most promising strain *P. citronellolis* N2 was used alone and together with oat and lupine plants for soil remediation of oil, including herbicide-treated oil-contaminated soil. Combined contamination was more toxic to plants and soil microorganisms. Bacterization stimulated the formation of chlorophyll and suppressed the synthesis of abscisic acid and malonic dialdehyde in plant tissues. The combined use of bacteria and oat plants most effectively reduced the content of hydrocarbons in the soil (including in the presence of herbicides). The results obtained can be used to develop new methods for bioremediation of soils with polychemical pollution.

## 1. Introduction

It is widely known that anthropogenic activities lead to large-scale pollution of the biosphere [1]. The most easily accessible and low-cost way to solve this global problem is bioremediation. It is based on the ability of living organisms (plants, microorganisms, and fungi) to eliminate or reduce the content of various pollutants to a safe level with minimal impact on the environment [2]. Decomposer microorganisms play a major role in this process, and recently, their combinations with plants have increasingly been used to remediate contaminated soils [3,4]. These combinations represent an example of a mutually beneficial partnership under conditions of abiotic stress. The surface of the root system serves as a site for the attachment of microorganisms; its exudates are a source of nutrients for bacteria (including bacteria that degrade organic pollutants). This leads to an increase in their numbers in the rhizosphere compared to the bulk of the soil [5,6]. In addition, roots secrete enzymes that degrade organic substrates (including pollutants) found in the soil [7]. Microorganisms, in turn, can stimulate plant growth through the biodegradation of xenobiotics that negatively affect plants, as well as through the formation of various biologically active substances (hormones, siderophores, etc.), improving mineral nutrition and increasing stress resistance and antagonism towards phytopathogens [8].

The most common environmental pollutants include oil, which is explained by the constantly growing demand for energy sources [9]. All processes associated with exploration, production, transportation, processing, storage, and use of hydrocarbons lead to their release into ecosystems [10]. These substances suppress the vital activity and reduce the diversity of plants and soil microorganisms, disrupt the structure and ecological balance of the soil, and also threaten human health [11,12,13,14]. Oil reduces soil fertility by changing its physical and chemical properties (reducing water conductivity and the availability of nitrogen, phosphorus, potassium, etc.), suppressing the vital activity of microorganisms. It has a phytotoxic effect on seed germination and plant growth (see review [15] and references therein). Therefore, it is not surprising that the issue of restoring oil-contaminated areas is an urgent issue all over the world. However, the use of bioremediation to clean soil with oil may be complicated by the presence of other pollutants in it. For example, crude oil spills can be accompanied by the release of heavy metals or various salts into ecosystems [16]. Oil-contaminated arable soils may contain residual amounts of chemical plant protection products (for example, herbicides) and heavy metals that accumulate as a result of the excessive use of mineral fertilizers and pesticides [17,18,19]. Each of these accompanying xenobiotics, in turn, has a negative impact on soil microbiota, as well as plant growth and development [20,21,22]. When present simultaneously, pollutants can enter into complex interactions, and the toxic effect they have on living organisms is usually enhanced [23,24]. Therefore, the use of bioremediation to eliminate the consequences of oil spills is associated, first of all, with the search and study of bacteria and plants that have the ability to degrade or reduce the toxicity of hydrocarbons and with resistance to additional pollutants. In addition, these microorganisms must decompose oil in the presence of other pollutants in the environment. The features of oil-contaminated soil bioremediation in the presence of heavy metals or salinity have been studied [25,26,27]. However, there is very little information on oil bioremediation of agricultural soils containing herbicides and on the effect of oil-degrading bacteria on plant growth and development under such combined contamination conditions [28,29]

The purpose of this study was to isolate oil-degrading microorganisms with growth-promoting properties and resistance to herbicides of various classes and to evaluate the effect of the most promising strain on plant growth in oil-contaminated soil and on the content of oil products in the soil, including in the presence of herbicides. We hypothesized that this strain would help plants overcome the effects of abiotic stress caused by oil, herbicides, and combinations thereof and also help cleanse soil of hydrocarbon contamination despite the presence of herbicides. Studying the characteristics of the interaction between microorganisms and plants under conditions of complex pollution with oil and herbicides will contribute to the development of methods for the biological purification of agricultural soils.

## 2. Results

### 2.1. Identification of Strains of Hydrocarbon-Oxidizing Microorganisms

From 42 isolates, 4 isolates were selected that grew most actively on liquid and agar Raymond medium with oil. The genera of these strains were determined using MALDI–TOF mass spectrometry, and their species were clarified by sequencing the nucleotide sequence of the 16S rRNA gene (Table S1).

Thus, it was found that strains C2 and H3.2 belong to the species *Acinetobacter calcoaceticus*, H4.1—*A. seifertii*, and N2—*Pseudomonas citronellolis*.

In addition to the strains isolated in this study, the strain *Acinetobacter courvalinii* UOM 35 was used in the experiments. This strain was isolated by the authors of this study from oil-contaminated arable soil in the Gafuriysky district of the Republic of Bashkortostan (Russia). The strain has a significant degree of biodegradation of oil in a liquid medium, as well as the ability for nitrogen fixation, phosphate mobilization, and IAA production [30]. However, its other PGP properties (plant growth-promoting properties), as well as resistance to herbicides of different classes and their effect on the properties of this strain have not been studied yet.

### 2.2. Properties of the Strains and the Effect of Oil and Herbicides on Them

All five strains were resistant to the presence of the herbicides Spetsnaz, Tapir, and Octapon extra in the concentration range of 1–10 mL(g)/L.

The strain *A. courvalinii* UOM 35 had the highest ability to degrade oil; in other microorganisms, it was lower and almost the same (71.8–74.1%). The addition of herbicides to the medium had different effects on the degree of oil biodegradation: Spetsnaz had no effect on this parameter; in the presence of Tapir, it decreased by 1.5–2.5 times. The herbicide Octapon extra turned out to be the most toxic. In *A. calcoaceticus* C2 and H3.2, the ability for biodegradation decreased by more than 6–7 times (Table 1). In general, the ability to decompose oil in the presence of herbicides in the *P. citronellolis* N2 strain was preserved to a greater extent than in other bacteria.

Bacteria have been studied for properties beneficial to plants, in particular, the ability to produce the phytohormone IAA. *A. calcoaceticus* C2 produced this auxin in amounts less than 150 ng/mL of culture liquid. Therefore, it did not participate in a further experiment to test the effect of oil and herbicides on the production of this compound. The remaining strains released IAA in amounts ranging from 320 (*A. calcoaceticus* H3.2) to 6025 ng/mL (*P. citronellolis* N2) (Table 2). The addition of oil or herbicides inhibited hormone synthesis in *A. calcoaceticus* H3.2. Octapon extra had a positive effect on its amount in the culture liquid of strains *A. courvalinii* UOM 35, *A. seifertii* H4.1, and *P. citronellolis* N2 (it increased 1.2–1.3 times). Other herbicides, on the contrary, suppressed the ability to form IAA, especially Tapir. When it was used, hormone production in *A. courvalinii* UOM 35 and *A. seifertii* H4.1 was less than 150 ng/mL. When Spetsnaz was added to the medium, the level of IAA in the bacterial culture liquid decreased by 1.2–2.3 times (Table 2).

All bacteria were capable of active growth on Ashby medium, which does not contain nitrogen sources. They were, at a minimum, oligonitrophils. In the presence of oil, the number of microorganisms either remained at the same level or increased. The addition of the herbicides Tapir and Spetsnaz to the medium also contributed to an increase in the number of cells of the strains by 1–2 orders of magnitude. But when Octapon extra was added, the bacterial titer decreased by 3–4 orders of magnitude compared to the control (Table 2).

The solubilization index of inorganic phosphate (SI) by microorganisms was 1.5–3.5. A high value of this parameter (SI > 3) was recorded for strains *A. calcoaceticus* C2 and *P. citronellolis* N2. Addition of pollutants to the medium resulted in a decrease in SI. Tapir had the most noticeable inhibitory effect on the phosphate-mobilizing activity of microorganisms, in the presence of which SI decreased by 1.3–3.0 times (Table 2).

When strains were grown in TSA medium, the color of filter paper soaked in picric acid did not change to brown. On CAS agar, no yellow-orange halo was detected around the wells containing the bacterial inoculum. This indicates that the studied microorganisms lack the ability to produce cyanide and siderophores.

Thus, based on the totality of the studied properties, the strain *P. citronellolis* N2 was selected for use in the experiment on removing oil from soil, including in the presence of herbicides. Additional pollutants had a less inhibitory effect on its ability to biodegrade oil, and its PGP properties (IAA production and phosphate mobilization) were preserved in the presence of pollutants to a greater extent than those of other studied bacteria.

### 2.3. Plant Growth Under the Influence of Oil, Herbicides, and Bacteria

**Figure 1:**
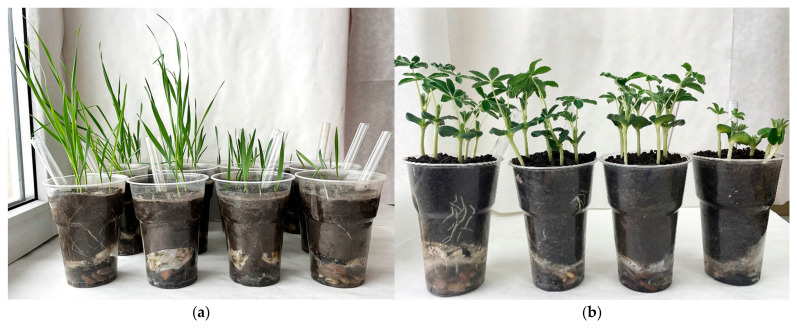
Oat plants (**a**) on the 14th day and lupine (**b**) on the 10th day of the experiment. From left to right: plants in clean soil (control) and in soil with Spetsnaz, Tapir, and Octapon extra.

**Figure 2:**
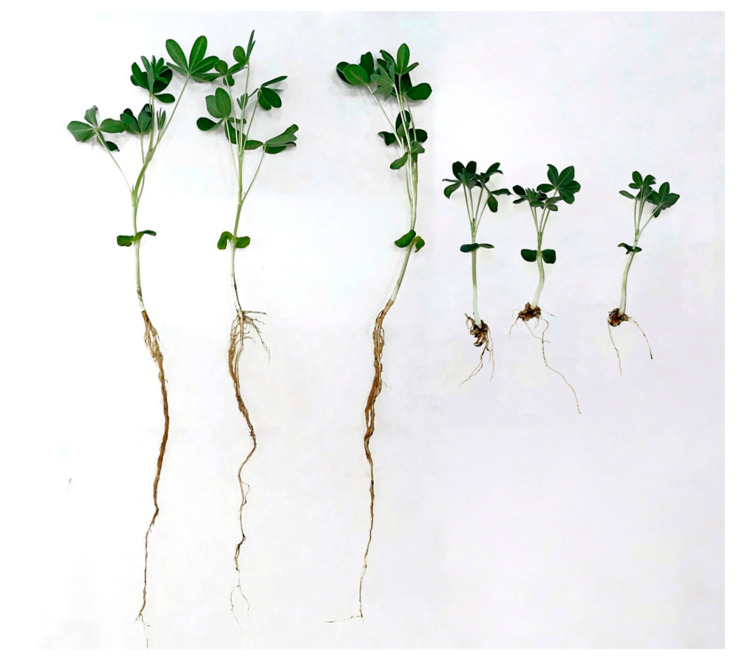
Lupine roots in clean soil (**left**) and in soil with Octapon extra (**right**) on the 16th day.

According to our preliminary experiments in soil in Petri dishes, oats and lupine showed resistance to oil and all herbicides used in this study [31]. However, in the pot experiment, the herbicides had an inhibitory effect on the length of roots and shoots of both plant species and also caused changes in plant morphology. At the same time, Octapon extra had the highest toxicity compared to other herbicides (Figure 1). In soil with this herbicide, oat and lupine seeds germinated with a delay of 1–2 days. Octapon extra sharply suppressed shoot growth and the development of the main and lateral roots (Figure 1 and Figure 2). In the soil with Octapon extra, most of the plants of both species (especially in the pots where the normal water distribution was disrupted due to oil contamination) died before the end of the experiment. Therefore, their morphological and biochemical parameters in the soil with this herbicide were not assessed. At the same time, the microbiological activity and hydrocarbon content in the soil treated with Octapon extra were determined.

**Figure 3:**
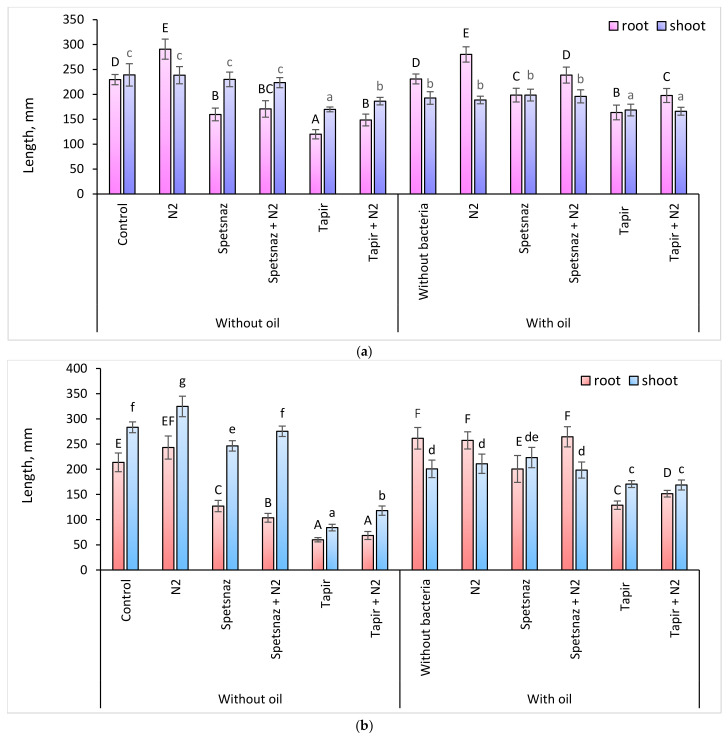
Root and shoot length of oats (**a**) and lupine (**b**) plants on the 21st day. Statistically different means values for each parameter are marked with different letters (for roots—capital letters, for shoots—lowercase letters; *p* ≤ 0.05, n = 15).

The application of herbicides led to a decrease in the root length in both plant species compared to plants in clean soil. This was especially noticeable when using Tapir: in oats and lupine, they became smaller by 3.6 and 1.9 times, respectively (Figure 3). The herbicides also had a negative effect on the length of shoots, although less significantly than on the roots. In oats, the shoot size decreased by 1.2 times when using Spetsnaz and by 3.4 times when treated with Tapir. No decrease in shoot height was observed in lupine under the influence of Spetsnaz, while Tapir caused its decrease by 1.4 times. The presence of oil in the soil led to an increase in the root length only of oats (by 1.2 times) and a decrease in the length of shoots (by 1.2–1.4 times) in both species compared to plants in clean soil. The simultaneous presence of oil and Spetsnaz inhibited the growth of the root system of oats and lupine (by 1.2–1.3 times) compared to plants in soil with oil (Figure 3).

Compared with oil, complex contamination with oil and Tapir led to a decrease in the length of oat and lupine roots by 2.0 and 1.4 times, respectively. At the same time, the length of the roots of both plants in the soil with oil and herbicides was greater than in the soil with only herbicides (1.6–2.1 times for oats and 1.2–1.4 times for lupine) (Figure 3). The length of oat shoots in the variants “oil” and “oil + Spetsnaz” did not differ significantly. The length of lupine shoots in the variants “oil” and “oil + Spetsnaz” was the same (192–198 mm) but was less than in clean soil or in the soil with Spetsnaz (230–239 mm). The length of the aboveground part in both species under the influence of Tapir and oil was lower than in plants in clean or oil-containing soil but greater (in oats) or equal to that (in lupine) in plants in soil with only the herbicide.

Inoculation of clean soil with *P. citronellolis* N2 increased the length of oat shoots by 15% (Figure 3a). In the presence of herbicides, bacterization also had a positive effect on the length of the aboveground part of oat plants, but did not lead to the growth of its roots. Against the background of oil, treatment with microorganisms did not change the length of oat shoots but increased the length of the roots with combined contamination. In lupine, the introduction of *P. citronellolis* N2 into clean soil lengthened the roots by 27%, as well as the underground and aboveground parts of plants in soil with Tapir (by 24 and 10%, respectively). In oil-containing soil with or without herbicides, the bacteria did not affect the length of lupine shoots but stimulated root growth (on average by 17%). In general, it can be noted that against the background of oil, both plant species experienced a decrease in shoot length and an elongation of roots. Tapir was more toxic to plants than Spetsnaz (especially for oats). Combinations of herbicides with oil had a less pronounced negative effect on the underground part of plants than when using only herbicides.

### 2.4. Content of Hormones in Plants

**Figure 4:**
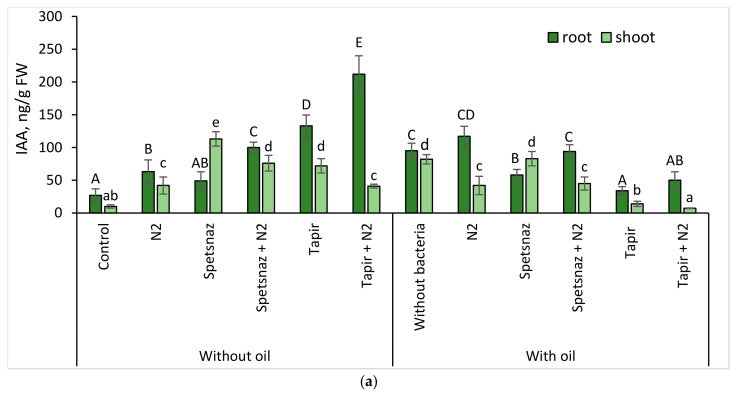

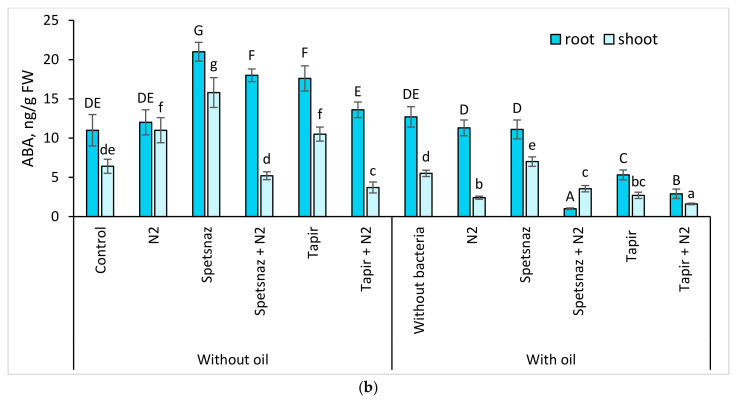
Indole-3-acetic acid (IAA) (**a**) and abscisic acid (ABA) (**b**) content in roots and shoots of oat plants on the 14th day. Statistically different means values for each parameter are marked with different letters (for roots—capital letters, for shoots—lowercase letters; *p* ≤ 0.05, n = 9).

Oil and herbicides, separately and together, increased the IAA content in oat plants (except for the variant oil + Tapir) (Figure 4a) and in the roots more significantly than in the shoots. However, in the presence of the herbicide Spetsnaz, including simultaneously with oil, its amount in the shoots was 2.3- and 1.4-times higher, respectively, than in the roots. This indicated a disruption in the transport of hormones from the shoots to the roots. Bacterization of clean soil led to an increase in the auxin level in the roots and shoots of oats. When introducing the strain *P. citronellolis* N2 into soil with various types of pollution, the tendency for the IAA content in the roots to increase compared to the untreated variants was maintained. The highest hormone content (212 ng/g) was found in the roots of inoculated plants in soil with Tapir. In the variants “oil + Spetsnaz” and “Spetsnaz”, the IAA concentration in the roots became higher than in the shoots as a result of the strain application.

The amount of ABA in the roots and shoots of oats with complex contamination was lower than in soil with only herbicides (Figure 4b). Bacterization of clean soil increased the hormone level in oat shoots. In contaminated soil, inoculation resulted in decreased ABA levels in oat roots and shoots.

**Figure 5:**
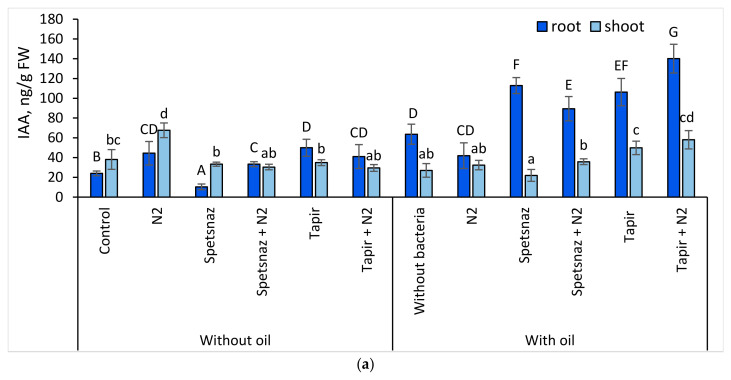

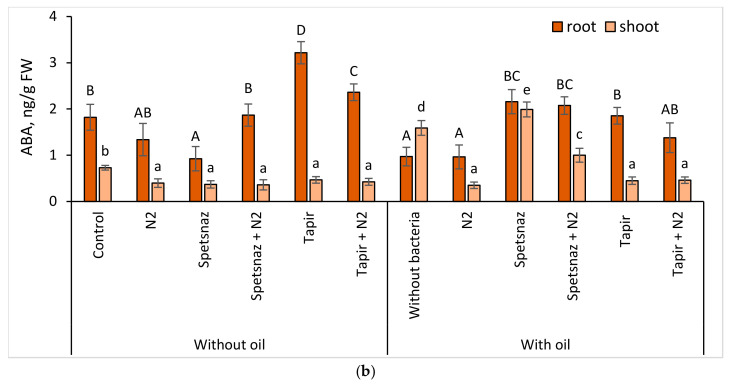
Indole-3-acetic acid (IAA) (**a**) and abscisic acid (ABA) (**b**) content in roots and shoots of lupine plants on the 14th day. Statistically different means values for each parameter are marked with different letters (for roots—capital letters, for shoots—lowercase letters; *p* ≤ 0.05, n = 9).

In general, the amount of IAA in the roots of lupine plants against the background of oil was higher than in the corresponding variants in soil without oil (Figure 5a). This had a positive effect on the length of the roots with combined pollution (an increase of 24.3–36.4%) compared to lupine plants in soil with only herbicides (Figure 3b). In the variant “Spetsnaz”, the concentration of IAA in the roots of lupine decreased by 2 times compared to the control. This resulted in a 1.4-fold reduction in the length of its roots. Lupine plants tried to overcome the inhibitory effect of Tapir (in soil without oil) by increasing the IAA content in the roots (by 2 times compared to the control), but this did not lead to the normalization of the growth of the underground part of the plants.

Treatment of clean soil with the auxin-synthesizing strain *P. citronellolis* N2 contributed to an increase in the level of IAA in the roots and shoots (by 1.8 times in both cases) and to the elongation of lupine roots (by 1.3 times) (Figure 3b and Figure 5a). In the variant “Spetsnaz + bacteria”, the amount of IAA in the roots increased by 3.3 times, but in the shoots, it did not change. In the variant “Spetsnaz + oil + bacteria”, the IAA level in the roots decreased, and in the shoots it increased. With combined contamination with oil and Tapir, the IAA content in the roots and shoots of lupine increased compared to plants in soil only with Tapir (by 2.1 and 1.4 times, respectively). The introduction of the strain into the soil with oil and Tapir further increased the hormone level in the roots.

In the soil without oil, a stable level of ABA was observed in the shoots of lupine (0.37–0.65 ng/g), but in the soil with oil and with oil and Spetsnaz, the hormone concentration in the aboveground part of the plants increased to 1.6–2 ng/g (Figure 5b). Inoculation of clean soil and in the variants “oil + Spetsnaz” and “Spetsnaz”, the accumulation of ABA in the shoots decreased compared to the corresponding options without the use of microorganisms.

In the roots of lupine, the highest level of ABA was found in plants in the soil with Tapir (3.22 ng/g). Bacterization reduced this parameter by 1.4 times (Figure 5b).

### 2.5. Pigment Complex and NBI

The total content of chlorophyll a and b in plants growing in the presence of oil alone was the lowest (20 and 13% less than the control values for oats and lupine, respectively) (Table 3). The presence of herbicides stimulated pigment production in oat leaves, and combined contamination had no inhibitory effect, or it was lower than against the background of oil. At the same time, Tapir significantly suppressed chlorophyll formation in lupine. Bacterization of clean and oil-contaminated soil, as well as soil containing a combination of pollutants, significantly increased the pigment content in oat plants (by 13–20%). In lupine, treatment with the strain slightly increased the chlorophyll level in clean soil and in the presence of oil or herbicides (by 5–8%).

The presence of oil in the soil led to a decrease in the amount of flavonoids in oat plants compared to the control, and each of the herbicides separately and a combination of Tapir with oil increased this parameter by 1.2–1.6 times (Table 3). The introduction of bacteria reduced the formation of these compounds only in those oat plants in the soil with Spetsnaz or Tapir (by 9%). In lupine, no significant trend in changing the flavonoid content under the influence of pollutants or microorganisms was observed.

NBI reflects a change in the ratio of chlorophyll and flavonoids and indicates the supply of nitrogen to plants. Under the influence of all types of pollution, it became 11–30% lower than the control value in oat plants (Table 3). A similar picture was observed in lupine. NBI decreased by 8–25% (except for pollution by oil and Spetsnaz). In both plant species, bacterization led to an increase in this parameter in both clean and contaminated soil.

### 2.6. Malondialdehyde Content

**Figure 6:**
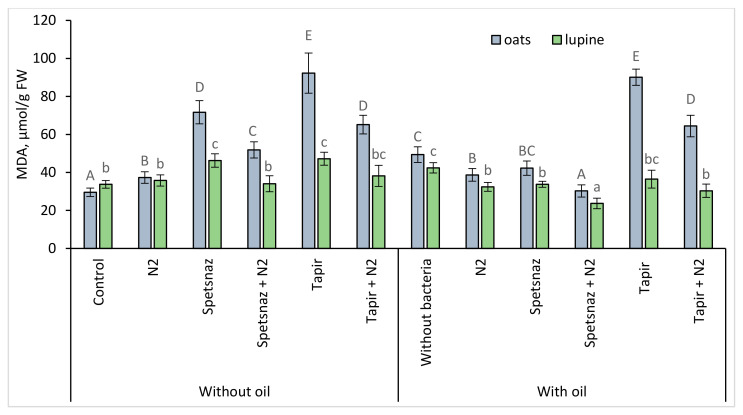
Malondialdehyde (MDA) content in plant leaves on the 14th day. Statistically different means values for each plant are marked with different letters (for oats—capital letters, for lupine—lowercase letters; *p* ≤ 0.05, n = 9).

Oil and herbicides, as well as their combinations, increased the malondialdehyde (MDA) content in oats and lupine (except for lupine plants that grew in soil with mixed contamination) (Figure 6). Bacterization of contaminated soil contributed to a decrease in this parameter, i.e., it reduced the stress level in both plant species. This was especially noticeable in lupine. In this plant, inoculation of soil contaminated with any pollutant or their combinations led to a decrease in the MDA level compared to the control value (33.75 μmol/g), and in the simultaneous presence of oil and Spetsnaz, the amount of MDA was lower than the control (23.69 μmol/g). In general, it should be noted that the amount of this compound in the leaves of oats was significantly higher than in lupine, especially if the herbicide Tapir was present in the soil. In addition, in oats, the introduction of microorganisms into clean soil also led to the accumulation of MDA, i.e., it was perceived by plants as a stress factor.

### 2.7. Number of Microorganisms of Different Groups in the Soil

The numbers of heterotrophic, oligonitrophilic, and hydrocarbon-oxidizing microorganisms in the bacterized soil were higher than in the soil where bioremediation was not used (Figure S1). Planting also contributed to an increase in the population of all three groups of microorganisms. Moreover, their number was greater in the lupine rhizosphere than in the root zone of oats. The combined use of plants and bacteria led to an even greater increase in the numbers of all three groups of microorganisms. This was especially noticeable in the inoculated soil with oats. Thus, depending on the type of pollution, the number of heterotrophic microorganisms in this soil increased by 2.5–15 times, hydrocarbon-oxidizing microorganisms by 4–50 times, and oligonitrophilic microorganisms by 3–60 times compared to similar variants in the soil where bioremediation was not used. The highest number of hydrocarbon-oxidizing microorganisms among all bioremediation options (bacteria, oats, lupine, bacteria + oats, and bacteria + lupine) was observed in the inoculated soil with oats, and the highest number of oligonitrophilic microorganisms was in the soil with bacteria and lupine.

For combined pollution with oil and herbicides, the number of microorganisms of all three analyzed groups was higher than for soil pollution with herbicides only (Figure S1).

### 2.8. Content of Total Petroleum Hydrocarbons in the Soil

**Figure 7:**
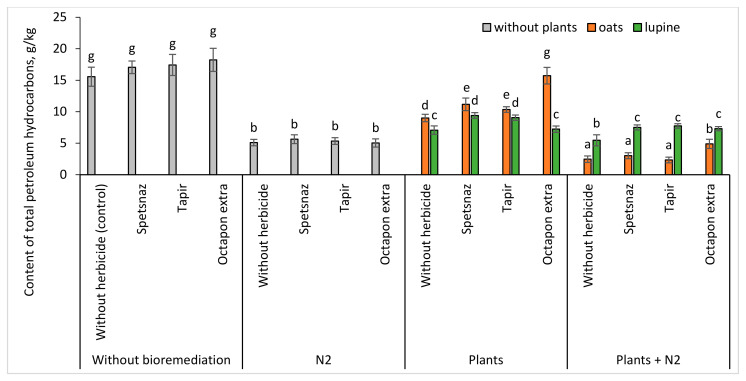
Content of total petroleum hydrocarbons in the soil on the 21th day. Statistically different mean values are indicated by different letters (*p* ≤ 0.05, n = 9).

As a result of soil self-purification in the absence of bioremediation, the hydrocarbon content in the soil decreased from 20 to 15.6–18.2 g/kg (Figure 7). The introduction of oil-degrading bacteria contributed to a decrease in this parameter to 5.0–5.6 g/kg. Planting was less effective than the use of microorganisms, but also led to a decrease in the amount of oil. In the case of combined contamination, lupine showed itself to be a more successful phytoremediator than oats (especially in the variant with oil and Octapon extra). But the lowest hydrocarbon content was achieved with the combined use of microorganisms and plants. At the same time, in the bacterized soil with oats, their quantity was lower than in the inoculated soil with lupine (2.3–4.9 and 5.5–7.7 g/kg, respectively).

## 3. Discussion

Recently, bioremediation has been increasingly recognized as the most appropriate method for eliminating the consequences of environmental pollution [8,32]. Its advantages are most noticeable where there is a need to restore large areas in situ, i.e., when the use of other methods is economically unprofitable. However, given that anthropogenic pollution is most often polychemical in nature [33], the possibilities of bioremediation may be limited by the presence of additional pollutants in the soil. These substances can significantly suppress the ability of microorganisms and plants to detoxify/degrade the main pollutant [34]. In this study, we focused on the search for oil-degrading microorganisms and the study of their growth-stimulating activity and resistance to xenobiotics such as herbicides. Residual amounts of chemical plant protection products are almost always present in agricultural soils, including those where oil spills have occurred. As a result of targeted screening, strains of bacteria of the genera *Acinetobacter* and *Pseudomonas* were isolated, and it was identified that they degraded oil by 72–74% (Table 1). Representatives of these genera are well known for their ability to biodegrade hydrocarbons [35,36]. We also included in further experiments the strain *A. courvalinii* UOM 35 isolated by the authors earlier, which has a significant potential for oil biodegradation (96%). All five strains had equally high resistance to the herbicides Spetsnaz, Tapir, and Octapon extra, the active ingredients of which belong to different classes of chemical compounds (Table 4).

A modern trend in environmental biotechnology is the isolation of pollutant-degrading bacteria and the creation of microbial–plant complexes on their basis. These associations provide a significant degree of purification due to the high adaptive potential and mutualistic relationships between plants and microorganisms [37]. Therefore, bacteria that decompose toxicants and enhance plant growth in contaminated soil (oil, in particular) are of great interest [38]. It is known that some *Pseudomonas* spp. and *Acinetobacter* spp. simultaneously possess these two characteristics [39,40]. All hydrocarbon-oxidizing bacteria participating in the experiments were studied for the presence of PGP properties. Most often, several features of microorganisms that have a positive effect on plant growth are noted, for example, the ability to improve the mineral nutrition of plants by increasing the availability of nitrogen and phosphorus. These macronutrients play a key role in the processes of growth and metabolism in plant cells [41,42]. An important factor in the growth-stimulating effect of bacteria is their ability to produce phytohormones, in particular, IAA. It enhances the division of plant cells and their elongation and differentiation, affects the architecture of roots and their exudation, promotes the absorption of nutrients, fruit development, and much more [43,44]. Inoculation with auxin-synthesizing bacteria helps plants cope with the stress caused by drought, salinity, oil, and herbicides [38,45,46,47]. All five strains studied were potentially capable of fixing atmospheric nitrogen and dissolving calcium orthophosphate. Four microorganisms produced IAA in the amount of 320–6025 ng/mL of the culture liquid. Moreover, among *Acinetobacter* spp., the maximum production of this hormone (2766 ng/mL) was noted in *A. seifertii* H4.1. Apparently, this is the first report of the presence of PGP properties in representatives of this species and, in particular, the ability to synthesize auxin (Table 2).

It was already mentioned above that stressful conditions can have a negative effect on the properties of strains. Therefore, it was interesting to study how herbicides affect the ability of hydrocarbon-oxidizing bacteria to degrade oil, as well as how the PGP properties of strains change under the influence of oil or herbicides. Octapon extra and Tapir sharply suppressed the process of oil biodegradation (Table 1). Perhaps this was due to their inhibitory effect on the activity of the enzymatic system responsible for the oxidation of hydrocarbons [48]. Oil had an ambiguous effect on the synthesis of IAA. In most strains, it suppressed this process and enhanced it only in *A. seifertii* H4.1. Both the stimulating/neutral and inhibitory effects of oil and oil products on the level of this hormone in the culture fluid of bacteria have been previously noted [38,49]. The herbicides Spetsnaz and Tapir reduced the level of IAA production. Information on a similar effect of pesticides of various classes on bacteria of the genera *Mesorhizobium*, *Pseudomonas*, *Burkholderia*, *Azotobacter*, *Bacillus*, and *Rhodococcus* was obtained earlier [50,51,52,53]. At the same time, Octapon extra enhanced the production of this phytohormone, probably due to the auxin-like structure of 2,4-D, which is the active substance of this herbicide (Table 2). Of all the pollutants, only Octapon extra led to a sharp decrease in the bacterial titer in a nutrient medium without a nitrogen source (Table 2). Apparently, the substances included in the herbicide inhibited the activity of the microorganisms’ nitrogenase complex [54]. This hypothesis is confirmed by the fact that when cultivating the same microorganisms on Raymond’s medium containing ammonium nitrate, the presence of this herbicide did not reduce the number of bacteria. Oil and herbicides caused inhibition of the ability to mobilize phosphate in the studied strains (Table 2). A similar negative effect was noted in [50,51,52]. Bacterial phosphate mobilization activity is usually associated with a decrease in pH, which can be caused by the secretion of low-molecular organic acids (gluconic, oxalic, acetic, etc.) or the action of phosphatases [55]. Possibly, pollutants inhibited the formation of these compounds. However, the work [56] described the strain *A. oleivorans* S4 as capable of simultaneous growth on a medium with hexadecane and the release of phosphates. In general, the results obtained in this study can help to expand the understanding of the toxic effect of oil and herbicides on the growth-stimulating properties of bacteria.

The strain *P. citronellolis* N2 retained its PGP properties and the ability to degrade oil in the presence of herbicides. Therefore, it was used in a pot experiment to clean up soil from oil in the presence of an additional pollutant (herbicide). Strains of this species degrade diesel fuel, PAHs, phenols, and pesticides (as part of a consortium) and have PGP properties [57,58,59,60,61]. The bacteria were used both separately and together with oat or lupine plants. As is known, residual amounts of herbicides can inhibit crop plants [62]. All herbicides used in this study inhibited the growth and development of plants of both species (reduction in the length of roots and shoots, changes in root architecture, yellowing of leaves, chlorosis, etc.) (Figure 1, Figure 2 and Figure 3). In the present study, the herbicide Octapon extra (active ingredient 2,4-D) had the highest toxicity (Figure 1 and Figure 2). Plants in soil with this preparation had a significant delay in growth and development and died before the end of the experiment. Despite the fact that 2,4-D has been used in agriculture for almost 80 years, the exact molecular mechanism of its action (including on non-target crops) has not been fully studied. Among other things, this may be due to excess ethylene production in response to 2,4-D treatment, which, in turn, stimulates the formation of ABA. The accumulation of this compound leads to the closure of stomata and a decrease in transpiration, which negatively affects the condition of plants [63]. Tapir suppressed both plants more significantly than Spetsnaz. This is probably due to its ability to accumulate in the soil and its high resistance to biological degradation [64].

Unlike herbicides, oil had a lesser negative effect on the morphometric parameters of plants of both species. Its presence did not inhibit the root length of lupine and increased it in oats (Figure 3). This may be due to an increase in the amount of IAA in the roots of both plant species in soil with hydrocarbons compared to control plants (Figure 4 and Figure 5), since one of the functions of this hormone is to stimulate root growth [44]. The ability of oil in small concentrations to stimulate plant growth has already been noted earlier [65]. Some authors explain this by the presence of substances with growth-regulating activity in its composition [66]. Since oil entering the soil reduces the availability of water and mineral nutrients for plants [67], maintaining root growth is an important response. It ensures the adaptation of plants to stressful conditions and promotes the colonization of the rhizosphere by bacteria. In addition, we observed a paradoxical effect of increasing the length of the underground part of plants in the soil with combined pollution compared to the same variants in the soil with only herbicides. Probably, oil somehow neutralizes the negative effect of herbicides, improving the living conditions of plants. The phenomenon that we discovered contradicts the information that with the simultaneous presence of pollutants, their negative impact on living organisms increases [23,24]. However, these studies do not consider the combined effect of oil and herbicides on plants.

The introduction of the *P. citronellolis* N2 strain partially compensated for the inhibitory effect of pollutants on plants. This is very important when carrying out soil cleanup using phytoremediation. The positive effect of bacterization is probably associated with the ability of this microorganism to destroy the pollutant and synthesize IAA. The effect of treatment with auxin-producing bacteria on the morphometric characteristics of oat plants was most noticeable where the soil was contaminated with herbicides or oil and herbicides. The introduction of *P. citronellolis* N2 into the soil with Spetsnaz or into the soil with Spetsnaz and oil shifted the balance of IAA content in oat plants in favor of the roots. The introduction of bacteria into the soil with Tapir led to the fact that the oat roots were observed to have the maximum concentration of IAA for this experiment. This indicates that the plants were attempting to restructure their hormonal system in such a way as to overcome the negative effect of Tapir on the roots (Figure 3a and Figure 4a).

An increase in the ABA content indicates that the plants are in unfavorable conditions for growth. Stress caused by the herbicides Spetsnaz and Tapir contributed to the accumulation of this hormone in the roots and shoots of oats. This led to inhibition of their length, especially noticeable in the soil with Tapir.

When bacterizing the soil with herbicides or with herbicides and oil, the level of IAA accumulation in the oat roots increased (in the case of contamination with oil and Tapir, this is unreliable), and the ABA content decreased. However, this affected the elongation of the root system only in the soil with combined contamination.

In lupine, the highest concentration of ABA was found in the roots of plants in the soil with Tapir. This resulted in their length being minimal compared to the plants in the control and in the soil with other types of pollution (Figure 3b and Figure 5b), although this herbicide is recommended for use on crops of leguminous plants, which include lupine. The introduction of bacteria into the soil with Tapir caused a decrease in the ABA content in lupine roots, which contributed to an increase in their length. The introduction of the oil-degrading strain reduced the amount of hydrocarbons in the soil. This led to a decrease in soil toxicity and, accordingly, a decrease in the ABA level and growth activation. It is known that oil and herbicides can suppress photosynthesis [68], which plays an important role in phytoremediation, providing oxygen to the process of pollutant destruction. Therefore, it was important to assess the effect of toxicants on photosynthesis parameters. For example, on the total chlorophyll content, since the amount of this pigment has a direct effect on the intensity of photosynthetic reactions [69]. Of all types of pollution, oil most significantly suppressed the formation of chlorophyll in plants of both species (Table 3). Inoculated plants tolerated the presence of oil, as well as oil and herbicides, more easily. This is indirectly evidenced by the increase in their pigment content compared to plants in soil with similar pollution, but without bacterization.

Antioxidants, substances capable of reacting with free radical compounds, are effective means of protecting plants from oxidative stress. These include flavonoids, a large class of low-molecular polyhydric phenols. The ratio between the amount of chlorophyll and flavonoids is clearly described by the nitrogen balance index (NBI). It is an indicator of changes in the N:C ratio in leaves and characterizes the nitrogen supply of plants [70,71]. The NBI values of plants in soil with contamination regardless of its type (except for lupine plants in soil with oil and Spetsnaz) were lower than those of control plants (Table 3). This indicates low nitrogen availability for plants of both species in the presence of pollutants. The introduction of the *P. citronellolis* N2 strain had a positive effect on the nitrogen status of plants.

Malondialdehyde is a product of lipid peroxidation. An increase in its concentration in cells indicates that plants are subject to severe oxidative stress [72]. All types of pollution led to an increase in this parameter in both plant species (Figure 6). The introduction of bacteria into the soil with pollutants contributed to a decrease in the amount of MDA in the leaves of plants of both species, sometimes even to control values or below.

All types of bioremediation (bacteria, plants, bacteria, and plants) significantly accelerated oil degradation, including in soil with combined pollution (Figure 7). The most significant reduction in the hydrocarbon content in the soil was achieved with the combined action of oat plants and microorganisms. Most likely, this is due to the high number of hydrocarbon-oxidizing bacteria in the rhizosphere of this plant (Figure S1) as a result of the positive effect of bacterization on the development of the root system. This improved soil aeration and, thus, contributed to the growth of aerobic microbiota [73]. But the main reason is probably that oat root exudates, compared to those of lupine, turned out to be more suitable for increasing the biomass of microorganisms of this group [5,74].

Having studied the available scientific literature, we believe that this work is one of the first to study the effect of an oil-degrading strain with growth-stimulating properties and resistance to herbicides on the morphometric and biochemical parameters of plants from the legume and cereal families under combined pollution with oil and herbicides. In addition, an assessment was made of the effectiveness of using this microorganism, including in combination with remediating plants, to clean up soil from oil in the presence of herbicides. In the future, we plan to study the ability of oil-degrading bacteria to degrade herbicides and how their introduction affects the rate of decomposition of herbicides and oil in the soil. The results obtained will contribute to the development of new effective methods for the bioremediation of soils contaminated with several pollutants.

## 4. Materials and Methods

### 4.1. Isolation and Identification of Strains of Hydrocarbon-Oxidizing Microorganisms

Strains of hydrocarbon-oxidizing microorganisms were isolated from samples of anthropogenically contaminated soils from the territory of the Russian Federation using the method of enrichment cultures [75]. We used Raymond’s mineral medium (composition (g/L): NH_4_NO_3_—2.0, MgSO_4_ × 7H_2_O—0.2, KH_2_PO_4_—2.0, Na_2_HPO_4_—3.0, CaCl_2_ × 6H_2_O—0.01, and Na_2_CO_3_—0.1) [76] and oil as the sole source of carbon and energy (4% *w*/*v*).

In all experiments with bacteria and plants, crude oil from the Yuzhno-Khylchuyuskoye oil field (Nenets Autonomous Okrug, Russia) was used. It is a light low-sulfur oil. Its characteristics are as follows: density 0.843 g/cm^3^, sulfur content 0.4%, resins—5.2%, asphaltenes—0.3%, and solid paraffins—2.0%. Group composition: methane hydrocarbons—48%, naphthenic hydrocarbons—42%, aromatic hydrocarbons—10%. The initial identification of strains was carried out by MALDI mass spectrometry using an Autoflex Speed device (Bruker Daltonics, Bremen, Germany) equipped with a time-of-flight analyzer as described in [77] (delay time is 350 ns, acceleration potential—20 kV the range of registered masses—2–20 kDa; spectral resolution ±2 Da; the resulting spectra for each strain preparation was obtained by summing the spectra registered in 10–15 points of the analyzed preparations at 500 hits of the laser). To determine the taxonomic affiliation of the strains, the Biotyper 3.0 program (Bruker Daltonics, Bremen, Germany) was used. To clarify the species of microorganisms, a fragment of the 16S rRNA gene sequence was sequenced. Total DNA was isolated according to the method described in [78]. Amplification of the 16S rRNA gene fragment was carried out with universal primers 27F (5′-AGAGTTTGATCTGGCTCAG-3′) and 1492R (5′-ACGGTACCTTGTTACGACTT-3′) [79]. PCR products were purified, and the subsequent sequencing reaction was performed using the Big Dye Terminator Cycle Sequencing Kit (Applied Biosystems, Carlsbad, CA, USA) according to the manufacturer’s instructions with the help of the ABI PRISM 3730 automatic sequencer (Applied Biosystems, Carlsbad, CA, USA). The search for 16S rRNA nucleotide sequences similar to the corresponding sequences of the studied strains was carried out using the EzBioCloud server (https://www.ezbiocloud.net, accessed on 28 March 2023).

### 4.2. Capacity for Oil Biodegradation, PGP Properties of Strains, and the Influence of Oil and Herbicides on These Characteristics

The hydrocarbon-oxidizing activity of the strains was assessed by the degree of destruction of the aliphatic fraction of oil using gas chromatography [80]. Bacteria were cultivated for 5 days in liquid Raymond medium with oil (4% *w*/*v*); then, the paraffin-naphthenic fraction of oil was extracted with hexane and analyzed on a gas chromatograph KRISTALLUX-4000 (NPF Meta-Chrome, LLC, Yoshkar-Ola, Russia) with a flame ionization detector and a Zebron™ ZB-1XT capillary column (Phenomenex, Torrance, CA, USA) (30 m × 0.53 mm × 2.65 µm; initial column temperature of 100 °C, heating rate of 5 °C/min, final temperature of 270 °C, and helium carrier gas). To assess the effect of herbicides on the biodegradation of oil, they were added to the medium (1% *w*/*v*) simultaneously with oil. The resistance of strains to herbicides was determined visually by the intensity of growth on nutrient agar (composition (g/L): peptone—10.0, yeast extract—3.0, glucose—1.0, NaCl—5.0, and agar–agar—15.0) [81] with the addition of various concentrations of herbicides (1–10 mL/L for liquid Octapon extra and Tapir and 1–10 g/L for dry Spetsnaz) after 5 days of cultivation at 28 °C. Selective herbicides produced in Russia, approved for use in the Russian Federation, were used (Table 4). These products are widely used in agriculture, and their active substances belong to different classes of chemical compounds.

To determine the amount of indolyl-3-acetic acid (IAA) in the culture liquid of the strains, bacteria were grown for 5 days on the nutrient medium (the composition is given above). The culture liquid was centrifuged at 8000 g followed by ultrafiltration through cassettes SARTOCON Slice with a pore diameter of 1 kDa (Sartorius Stedim Biotech GmbH, Goettingen, Germany). Ultrafiltrates were analyzed on an LC-20 Prominence HPLC system with an SPD-M20A diode array detector (Shimadzu, Kyoto, Japan) (Zorbax-ODS 5 μm column (250 × 4.6 mm) (Agilent Technologies, Santa Clara, CA, USA); solvent ratio 0.1% acetic acid in water: acetonitrile, 20:80; wavelength of 279 nm). The IAA content was determined using a calibration curve constructed using a standard (Sigma-Aldrich, St. Louis, MO, USA) in the concentration range of 10–10,000 ng/mL [82]. To study the effect of oil and herbicides on IAA production, they were added to the medium in an amount of 1% *w*/*v*.

The number of bacteria on a medium without a nitrogen source, including in the presence of oil and herbicides, was determined by growth rates on selective Ashby medium (composition (g/L): mannitol—20.0, K_2_HPO_4_—0.2, MgSO_4_ × 7H_2_O—0.2, NaCl—0.2, K_2_SO_4_—0.1, and CaCO_3_—5.0) [75], which does not contain protein or mineral nitrogen. Oil and herbicides were added to the medium in a volume of 1%. Bacteria were cultured for 72 h. The strains were considered active if the number of their cells increased by no less than 3 orders of magnitude with an initial number of 10^3^ CFU/mL (colony forming units).

The ability of microorganisms to dissolve inorganic phosphates was determined on Pikovskaya medium (composition (g/L): glucose—10.0, (NH_4_)_2_SO_4_—0.5, NaCl—0.2, MgSO_4_ × 7H_2_O—0.1, KCl—0.2, MnSO_4_ × 7H_2_O—0.5, FeSO_4_ × 7H_2_O—0.5, yeast extract—0.5, and agar–agar 15.0) [83] with the addition of tricalcium phosphate. Bacteria were cultivated for 10 days at 28 °C. For this purpose, the solubilization index (SI) was calculated as follows: the ratio of the diameter of the clearing zone around the bacterial colony (mm) to the diameter of the colony (mm). If the SI value was less than 2, equal to 2–3, or greater than 3, then it was considered that the isolate had a low, medium, or high solubilization potential, respectively [84]. To study the effect of pollutants on the phosphate-mobilizing activity of bacteria, herbicides were added to the medium (1% *w*/*v*) before pouring into Petri dishes, and oil was applied to the surface of the solidified agar medium (250 μL).

The production of hydrocyanic acid (HCN) was determined as described in [85]. Microorganisms were grown on tryptic soy agar (Difco Laboratories, Detroit, MI, USA) supplemented with glycine (4.4 g/L). Strips of filter paper soaked in a solution of picric acid (0.5% picric acid and 2% sodium carbonate) were placed in the lid of a Petri dish, after which it was turned upside down and incubated for 4 days at 28 °C. The brown color of the paper indicated the production of HCN by the strains.

Siderophore production was determined on chromasurol S (CAS) agar [86]. A well with a diameter of 5 mm was made in the center of the CAS agar plate, into which the bacterial inoculum was added and incubated for 7 days at 28 °C. The appearance of a yellow-orange halo around the wells indicated the production of siderophores by the strains.

### 4.3. Plant Growth Conditions and Treatments

The microcosm-scale experiment was carried out according to a completely randomized design with the following parameters:

(1) Seven types of pollution: oil, herbicides Spetsnaz, Tapir, Octapon extra, and combinations of oil with one of the herbicides. Pollutants were added to a vessel (volume 0.5 L and height 14.5 cm) with 400 g of a soil–sand mixture (9:1) in the following quantities: oil—20 g/kg, Octapon extra and Tapir—20 μL/kg, and Spetsnaz—0.5 mg/kg soil. Doses of herbicides were increased by 2 times compared to manufacturers’ recommendations;

(2) Two types of plants: oats (*Avena sativa* L.,) of the Rysak variety (*Poaceae*)—6 sprouted seeds per 1 vessel—and white lupine (*Lupinus albus* L.) of the Dega variety (*Fabaceae*)—5 sprouted seeds per 1 vessel. These plants have already been used as phytoremediants [87,88,89,90,91]. Previously, we obtained data that they were resistant to oil and herbicides of various classes [31];

(3) Six different tests for each plant and type of pollution were conducted: (1) control test (clean soil with some plant without pollutants); (2) tests with the addition of strain *P. citronellolis* N2 to clean soil without contaminants in which plants of any species are planted; (3) tests using soil containing petroleum or its combination with any herbicide; (4) tests using soil containing any contaminant or combination thereof in which plants of any species are planted; (5) tests adding *P. citronellolis* N2 to soil containing oil or its combination with any herbicide; (6) tests involving the addition of *P. citronellolis* N2 to soil containing any contaminant or combination thereof, in which plants of any species are planted.

Tests using plants were performed in five replicates, and for the remaining tests, three replicates were performed for a total of 202 microcosms. Immediately before planting oat or lupine seedlings, the soil was moistened with either 50 mL of tap water or 50 mL of diluted culture liquid of the *P. citronellolis* N2 with a titer of 10^6^ CFU/mL. After 7 days, the soil in the corresponding beakers was again inoculated with bacteria. Plants were grown for 21 days at a temperature of 22–24 °C under controlled lighting (photon flux intensity of 240 µmol m^−2^ s^−1^ PAR; 14 h photoperiod). Humidity was maintained at 60% of the total soil moisture capacity.

On the 14th day of the experiment, the content of hormones in the shoots and roots of plants, the concentration of chlorophyll, flavonoids, and malondialdehyde (MDA) in the leaves of plants, and the nitrogen balance index (NBI) of plants was analyzed. After the end of the experiment, the morphometric parameters of plants (length of shoots and roots), the number of some groups of microorganisms, and the content of the hydrocarbons in the soil were assessed.

### 4.4. Content of Hormones in Plants

The content of hormones IAA and abscisic acid (ABA) in plant tissues were determined. The youngest leaves were used for chemical tests. To isolate phytohormones, shoots and roots were homogenized and extracted with 80% ethanol. Extraction of ABA and IAA from aliquots of aqueous residues was performed with diethyl ether, according to the modified method, as described by [92]. Hormones were immunoassayed using the corresponding specific antibodies.

### 4.5. Pigment Complex and NBI

The content of chlorophyll (*a* + *b*), flavonoids and the NBI in the leaves was measured using a DUALEX SCIENTIFIC+ device (FORCE-A, Centre Universitaire Paris-Sud, France) according to the manufacturer’s recommendations.

### 4.6. Malondialdehyde Content

Membrane lipid peroxidation was assayed as the amount of MDA. Fresh wheat leaves were homogenized in 10% trichloroacetic acid and then centrifuged at 10,000× *g* rpm. The amount of MDA in the extract was determined by the spectrophotometric method, by reaction with thiobarbituric acid [93]. Measurements were carried out in three biological and three analytical repetitions.

### 4.7. Number of Microorganisms of Different Groups in the Soil

In order to estimate the microbial counts in soil, a serial dilution of a soil suspension was used. The number of heterotrophic microorganisms was measured by application to the nutrient agar. For measuring the number of petroleum degrading bacteria, we used Raymond agar, supplemented with 0.1 g of sterile diesel fuel as the only source of carbon, smeared on the agar surface of each plate. To measure the number of oligonitrophilic microorganisms, we used Ashby medium.

### 4.8. Content of Total Petroleum Hydrocarbons in the Soil

Total petroleum hydrocarbons (TPHs) in the soil samples were measured using the EPA 3540C method. A total of 10 g of soil samples were packed in filter paper and extracted in a Soxhlet extractor with 300 mL of hexane for 8 h with six extraction cycles per hour. The extraction product was transferred to a glass column filled with glass wool and Na_2_SO_4_ to remove any water it contained. The extract was collected in a flask for subsequent evaporation of the solvent using a rotary evaporator Rotavapor R-100 (Buchi Labor-technik AG, Flawil, Switzerland) until a final volume of 2 mL was reached. The concentrated solution was poured into a pre-weighed glass beaker and dried until a constant weight was reached. TPHs present in the samples were then quantified by gravimetric analysis with a weighing accuracy of up to 0.1 mg.

### 4.9. Statistical Analysis

The data were processed using Statistica (Statsoft) software (version 10). In figures and tables, data are presented as mean ± standard error. The significance of differences was assessed by ANOVA followed by Duncan’s test (*p* ≤ 0.05).

## 5. Conclusions

In this study, strains of hydrocarbon-oxidizing bacteria of the genera *Pseudomonas* and *Acinetobacter* with PGP properties (IAA production and phosphate mobilization) and resistance to herbicides, the active substances of which belong to different classes of chemical compounds, were isolated and identified. The presence of herbicides based on 2,4-D and imazethapyr in the medium suppressed the biodegradation of oil, and the herbicide based on tribenuron-methyl did not affect this process. Oil and herbicides reduced the ability to dissolve phosphates and stimulated or inhibited the production of IAA. The combined use of the bacteria *P. citronellolis* N2 and oat plants significantly reduced the oil content in the soil (including in the presence of herbicides) compared to bacteria, oat plants, or lupine plants separately, due to the high number of hydrocarbon-oxidizing microorganisms in the oat rhizosphere. Combined pollution had a less pronounced toxic effect on both plant species and soil microbiota compared to pollution with herbicides alone. Bacterization helped plants overcome stress caused by the presence of oil, herbicides, and their combinations in the soil due to an increase in chlorophyll formation and a decrease in the amount of MDA and ABA.

## Figures and Tables

**Table 1:** Biodegradation of oil (%) and the effect of herbicides on it.

Strain	Oil	Oil + Spetsnaz	Oil + Tapir	Oil + Octapon Extra
UOM 35	95.9 ± 6.2 ^c^*	91.7 ± 5.8 ^c^	38.3 ± 3.2 ^b^	30.1 ± 3.2 ^a^
C2	74.1 ± 2.8 ^c^	73.0 ± 4.5 ^c^	35.9 ± 2.4 ^b^	10.2 ± 0.7 ^a^
H3.2	71.8 ± 5.1 ^c^	73.9 ± 3.7 ^c^	30.6 ± 2.3 ^b^	10.7 ± 0.4 ^a^
H4.1	72.7 ± 3.5 ^c^	70.1 ± 5.1 ^c^	36.3 ± 3.0 ^b^	21.7 ± 1.0 ^a^
N2	72.6 ± 3.7 ^c^	73.0 ± 4.1 ^c^	47.6 ± 2.7 ^b^	39.5 ± 1.9 ^a^

Statistically different means for each strain in each line are indicated by different letters (*p* ≤ 0.05). *—data from [30]. Bacteria were cultivated for 5 days in liquid Raymond medium with oil (4% *w*/*v*). Herbicides (1% *w*/*v*) were added to the medium simultaneously with the oil.

**Table 2:** PGP properties of microorganisms and the effect of oil and herbicides on them.

Strain	Control (Medium Without Pollutant)	Oil	Spetsnaz	Octapon Extra	Tapir
IAA production ^1^, ng/mL culture liquid					
UOM 35	658 ± 37 ^c^*	332 ± 29 ^b^	283 ± 37 ^b^	798 ± 52 ^d^	<150 ^a^
C2	<150	n/d	n/d	n/d	n/d
H3.2	320 ± 23 ^b^	<150 ^a^	<150 ^a^	<150 ^a^	<150 ^a^
H4.1	2766 ± 152 ^c^	4149 ± 223 ^e^	2387 ± 158 ^b^	3545 ± 208 ^d^	<150 ^a^
N2	6025 ± 315 ^c^	5878 ± 352 ^c^	3867 ± 254 ^b^	7133 ± 375 ^d^	3261 ± 215 ^a^
Number of microorganisms on a medium without a nitrogen source ^2^, CFU/mL					
UOM 35	10^6^ *	10^6^	10^7^	10^2^	10^7^
C2	10^6^	10^7^	10^7^	10^2^	10^7^
H3.2	10^6^	10^7^	10^7^	10^2^	10^7^
H4.1	10^6^	10^7^	10^8^	10^2^	10^8^
N2	10^6^	10^7^	10^8^	10^3^	10^8^
The phosphate solubilization index (SI) ^3^					
UOM 35	2.5 ± 0.1 ^b^*	1.8 ± 0.1 ^a^	1.7 ± 0.1 ^a^	1.6 ± 0.1 ^a^	1.6 ± 0.2 ^a^
C2	3.3 ± 0.2 ^d^	2.1 ± 0.1 ^b^	2.6 ± 0.2 ^c^	1.2 ± 0.1 ^a^	1.1 ± 0.2 ^a^
H3.2	1.5 ± 0.1 ^b^	1.3 ± 0.2 ^ab^	1.5 ± 0.1 ^b^	1.4 ± 0.2 ^ab^	1.1 ± 0.1 ^a^
H4.1	2.9 ± 0.3 ^b^	1.8 ± 0.1 ^a^	2.5 ± 0.2 ^b^	2.5 ± 0.2 ^b^	1.6 ± 0.1 ^a^
N2	3.5 ± 0.3 ^c^	2.3 ± 0.2 ^ab^	2.5 ± 0.1 ^b^	2.4 ± 0.2 ^ab^	2.1 ± 0.1 ^a^

Strain cultivation conditions: “^1^”—5 days, nutrient broth; “^2^”—3 days, Ashby medium; “^3^”—10 days, Pikovskaya agar. Oil and herbicides were added to the medium in an amount of 1% *w*/*v*. Statistically different means for each strain in each line are indicated by different letters (*p* ≤ 0.05). n/d —not determined due to low production ability. *—data from [30].

**Table 3:** Content of chlorophyll (*a* + *b*), flavonoids, and NBI in leaves.

Variants of Treatments		Oats			Lupine		
		Chlorophyll, (μg/cm^2^)	Flavonoids, (μg/cm^2^)	NBI	Chlorophyll, (μg/cm^2^)	Flavonoids, (μg/cm^2^)	NBI
Without oil	Control	25.4 ± 0.4 ^c^	0.61 ± 0.02 ^b^	43.1 ± 1.3 ^d^	37.2 ± 0.5 ^c^	0.27 ± 0.02 ^ab^	141.5 ± 2.5 ^d^
	N2	30.0 ± 0.8 ^e^	0.60 ± 0.02 ^b^	50.4 ± 1.4 ^e^	39.3 ± 0.9 ^d^	0.25 ± 0.02 ^ab^	152.7 ± 3.1 ^e^
	Spetsnaz	28.9 ± 1.0 ^de^	0.76 ± 0.01 ^d^	38.5 ± 0.8 ^c^	37.0 ± 0.5 ^c^	0.29 ± 0.02 ^b^	127.3 ± 2.3 ^c^
	Spetsnaz + N2	29.9 ± 0.9 ^e^	0.69 ± 0.02 ^c^	43.7 ± 1.1 ^d^	38.8 ± 0.5 ^d^	0.29 ± 0.01 ^b^	133.4 ± 2.6 ^c^
	Tapir	28.3 ± 1.0 ^de^	0.95 ± 0.03 ^f^	30.4 ± 0.7 ^a^	32.6 ± 0.5 ^a^	0.30 ± 0.01 ^b^	106.8 ± 1.5 ^a^
	Tapir + N2	27.5 ± 0.8 ^d^	0.86 ± 0.02 ^e^	32.7 ± 0.5 ^b^	35.0 ± 0.5 ^b^	0.29 ± 0.01 ^b^	119.8 ± 2.2 ^b^

With oil	Without bacteria	20.2 ± 0.5 ^a^	0.52 ± 0.02 ^a^	37.4 ± 1.0 ^c^	32.3 ± 0.4 ^a^	0.25 ± 0.02 ^ab^	129.4 ± 2.8 ^c^
	N2	24.2 ± 0.6 ^b^	0.52 ± 0.01 ^a^	43.9 ± 1.0 ^d^	34.8 ± 0.7 ^b^	0.22 ± 0.01 ^a^	154.2 ± 2.8 ^e^
	Spetsnaz	23.5 ± 0.6 ^b^	0.61 ± 0.01 ^b^	37.8 ± 0.7 ^c^	35.8 ± 0.4 ^b^	0.25 ± 0.02 ^ab^	143.9 ± 2.1 ^d^
	Spetsnaz + N2	27.3 ± 0.7 ^d^	0.60 ± 0.02 ^b^	42.8 ± 1.1 ^d^	35.3 ± 0.3 ^b^	0.23 ± 0.01 ^a^	154.4 ± 3.1 ^e^
	Tapir	25.5 ± 0.5 ^c^	0.69 ± 0.02 ^c^	37.0 ± 0.9 ^c^	33.9 ± 1.4 ^ab^	0.27 ± 0.02 ^ab^	130.0 ± 2.7 ^c^
	Tapir + N2	28.9 ± 1.0 ^de^	0.64 ± 0.02 ^bc^	44.1 ± 1.0 ^d^	34.4 ± 0.9 ^b^	0.24 ± 0.01 ^a^	143.1 ± 2.7 ^d^

Measurements were carried out on the 14th day of the experiment. Statistically different means values for each parameter are marked with different letters (*p* ≤ 0.05, n = 9). The values in each column are compared.

**Table 4:** Information on herbicides.

Product	Spetsnaz	Tapir	Octapon Extra
Manufacturer	Euroagrochemicals LLC, Ufa, Russia	Agro Expert Group, LLC, Volgograd, Russia	AHK-AGRO, LLC, Ufa, Russia
Preparative form	water-dispersible granules	water-soluble concentrate	emulsion concentrate
Active substance, empirical formula, content of the active substance	tribenuron-methyl, C_15_H_17_N_5_O_6_S 750 g/kg	imazetapir, C_15_H_19_N_3_O_3_, 100 g/L	2,4-dichlorophenoxyacetic acid (2,4-D), C_8_H_6_Cl, 500 g/L
Class of chemical compounds	sulfonylurea	imidazolinones	aryloxyalkano-carboxylic acids
Crops	cereals	legumes	cereals
Object of influence (weeds)	dicotyledons	dicotyledons, cereal	dicotyledons

## Data Availability

The data presented in this study are available in the graphs and tables provided in this manuscript.

## Supplementary Materials

The following supporting information can be downloaded at https://www.mdpi.com/article/10.3390/plants13243560/s1: Figure S1: The number of heterotrophic, oligonitrophilic, and hydrocarbon-oxidizing microorganisms in soil with different variants of bioremediation. Table S1: Source of isolates and their identification.
