# Supplementary figures and images for: Effect of Herbicide-Resistant Oil-Degrading Bacteria on Plants in Soil Contaminated with Oil and Herbicides
Source: Plants (Basel). 2024 Dec 20;13(24):3560. doi: 10.3390/plants13243560 (PMC11678539; doi:10.3390/plants13243560)

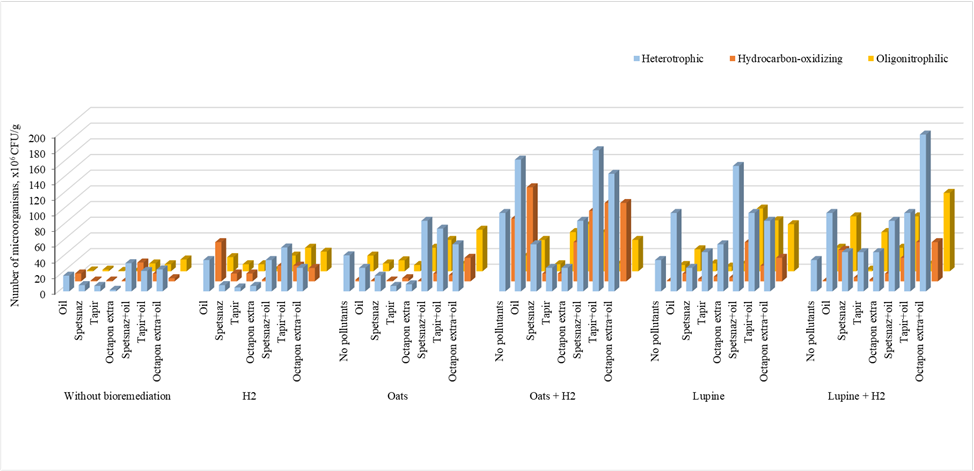

Supplement: Supplementary file 1 [file plants-13-03560-s001.zip › Fig. S1_The number of microorganisms in the soil under different bioremediation options.tiff]

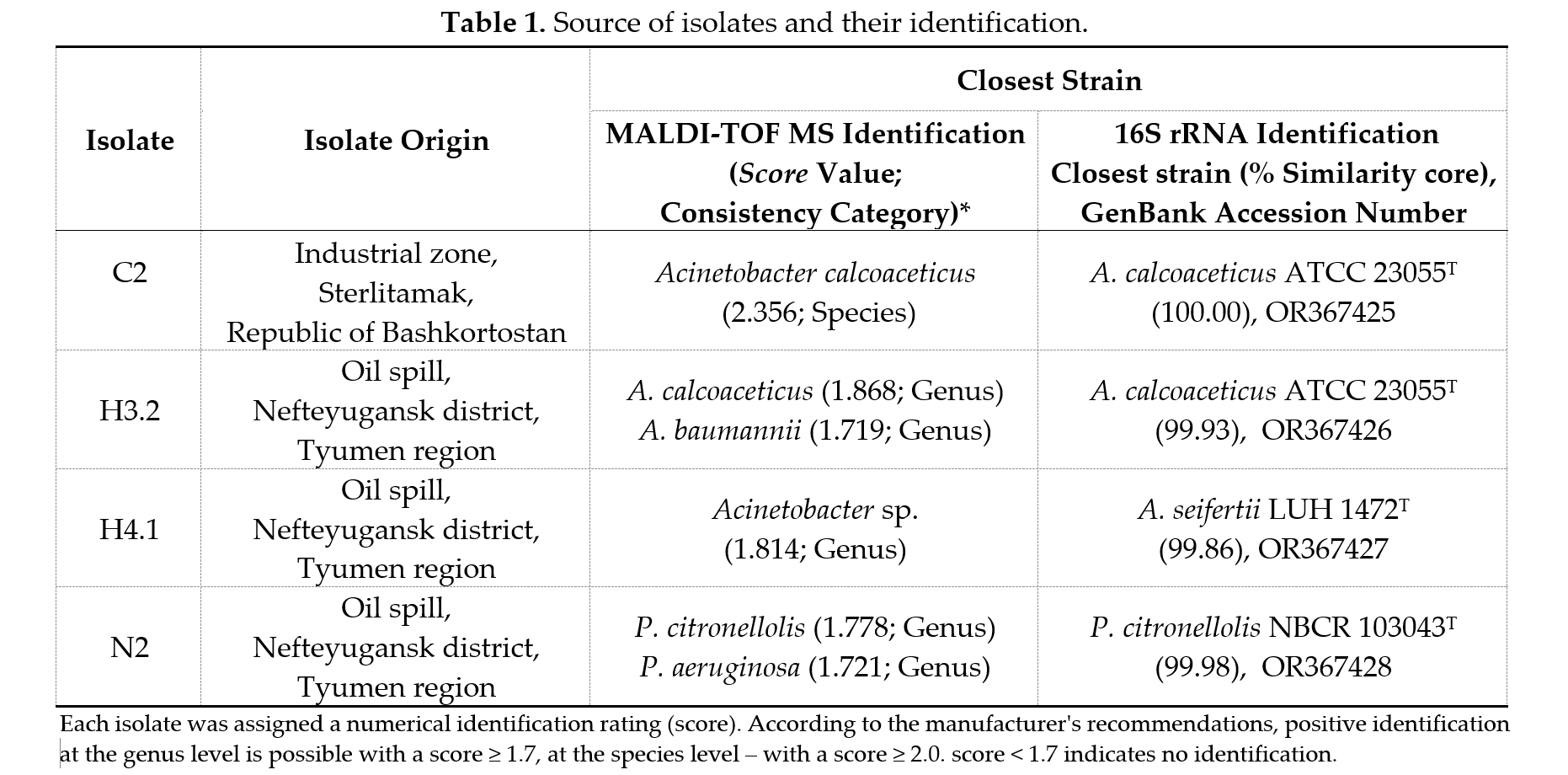

Supplement: Supplementary file 1 [file plants-13-03560-s001.zip › Table S1. Source of isolates and their identification.tif]
